# A RESEARCH AGENDA TO ADVANCE FAMILY CAREGIVERS' FINANCIAL SECURITY

**DOI:** 10.1093/geroni/igac059.1132

**Published:** 2022-12-20

**Authors:** Rani Snyder, Eileen Tell

**Affiliations:** The John A. Hartford Foundation, New York, New York, United States; ET Consulting, LLC, Belmont, Massachusetts, United States

## Abstract

The research supporting the RAISE Family Caregiver Advisory Council engaged a broad range of stakeholders who were committed to supporting actions that make a difference to family caregivers’ financial security, with many focusing on actions that sustain continued employment; 103 different organizations participated, including employer representatives. Participants strongly agreed on the need for more data. On a broad level, there was agreement on federal-level coordination regarding data collection on elements specific to family caregivers across federal agencies. In addition, they called for better information on the return on investment of varying strategies for workplace supports for employers, the added value of caregiver employees, and evidence regarding best practices in supporting family caregivers, with a goal of making a business case for family caregiver workplace supports.

